# Expression of Genes Related to Prostaglandin Synthesis or Signaling in Human Subcutaneous and Omental Adipose Tissue: Depot Differences and Modulation by Adipogenesis

**DOI:** 10.1155/2014/451620

**Published:** 2014-11-11

**Authors:** Andréanne Michaud, Nicolas Lacroix-Pépin, Mélissa Pelletier, Marleen Daris, Laurent Biertho, Michel A. Fortier, André Tchernof

**Affiliations:** ^1^Endocrinology and Nephrology, CHU de Québec, 2705 Laurier Boulevard (R-4779), Québec, QC, Canada G1V 4G2; ^2^Department of Nutrition, Laval University, 2425 Rue de l'Agriculture, Québec, QC, Canada G1V 0A6; ^3^Reproduction and Biology, CHU de Québec, 2705 Laurier Boulevard, Québec, QC, Canada G1V 4G2; ^4^Gynecology Unit, CHU de Québec, 2705 Laurier Boulevard, Québec, QC, Canada G1V 4G2; ^5^Department of Surgery, Institut Universitaire de Cardiologie et de Pneumologie, 2725 Chemin Sainte-Foy, Québec, QC, Canada G1V 4G5

## Abstract

*Objectives*. (1) To examine depot-specific PGE_2_ and PGF_2*α*_ release and mRNA expression of enzymes or receptors involved in PG synthesis or signaling in human adipose tissues; (2) to identify changes in expression of these transcripts through preadipocyte differentiation; and (3) to examine associations between adipose tissue mRNA expression of these transcripts and adiposity measurements. *Methods*. Fat samples were obtained surgically in women. PGE_2_ and PGF_2*α*_ release by preadipocytes and adipose tissue explants was measured. Expression levels of mRNA coding for enzymes or receptors involved in PG synthesis or signaling were measured by RT-PCR. *Results*. Cultured preadipocytes and explants from omental fat released more PGE_2_ and PGF_2*α*_ than those from the subcutaneous depot and the corresponding transcripts showed consistent depot differences. Following preadipocyte differentiation, expression of PLA2G16 and PTGER3 mRNA was significantly increased whereas COX-1, COX-2, PTGIS, and PTGES mRNA abundance were decreased in both compartments (*P* ≤ 0.01 for all). Transcripts that were stimulated during adipogenesis were those that correlated best with adiposity measurements. *Conclusion*. Cells from the omental fat compartment release more PGE_2_ and PGF_2*α*_ than those from the subcutaneous depot. Obesity modulates expression of PG-synthesizing enzymes and PG receptors which likely occurs through adipogenesis-induced changes in expression of these transcripts.

## 1. Introduction

The metabolic and endocrine functions of adipose tissues are altered in obesity [1]. Excess nonesterified fatty acids, immune cell infiltration, and the release of inflammatory cytokines have been proposed as the link between obesity, insulin resistance, and type 2 diabetes [2, 3]. In chronic overfeeding conditions, adipose tissue expansion occurs through adipocyte hypertrophy and the differentiation of preadipocytes to mature, lipid-storing adipocytes (hyperplasia) [4, 5]. The latter process involves important changes in gene expression, such as proadipogenic transcription factors peroxisome proliferator-activated receptor (PPAR) *γ* and CCAAT/enhancer-binding proteins (C/EBPs) [6, 7].

Prostaglandins (PGs) are lipid mediators secreted by various cell types in adipose tissue [8, 9] and they appear to be involved in the regulation of inflammation and adipocyte functions [10]. PGs are derived from arachidonic acid (AA), which is liberated by the phospholipid membrane through the activity of phospholipase A2 (PLA2) [11]. Jaworski et al. previously demonstrated that AdPLA (PLA2G16) is the main PLA2 enzyme in adipose tissue of mice [12]. AA is consecutively converted into PGG_2_ and PGH_2_ by the action of the two PGH synthases (PTGS), the constitutive cyclooxygenase- (COX-) 1, or the inducible COX-2 [13]. PGH_2_ is the common substrate for specific PG synthase enzymes that synthesize PGs [11]. PGD synthase (PTGDS) catalyzes the isomerization of PGH_2_ to PGD_2_, while prostacyclin synthase (PTGIS) catalyzes the isomerization of PGH_2_ to PGI_2_ [11]. The formation of PGE_2_ from PGH_2_ is catalyzed by PGE synthases (PTGES). Three forms of PGE synthase have been identified, including cytosolic PGE synthase (cPGES), which is constitutively and ubiquitously expressed [14], and the two membrane-bound PGE synthases (mPGES-1 and mPGES-2) [14, 15]. Fujimori et al. demonstrated that mPGES-1 acts as the main PGE_2_ synthase in 3T3-L1 cells [16]. PGF_2*α*_ is synthesized by enzymes of the aldo-keto reductase (AKRs) family through various pathways [17, 18]. The first PGF synthase identified in mammals was AKR1C3 [19]. However, we demonstrated for the first time in models including human preadipocytes as well as bovine and human endometrium that enzymes of the AKR1B family also show PGF synthase activity [8, 20–22]. Specifically, we recently established in human adipose tissue that preadipocytes from the omental fat compartment released more PGF_2*α*_ in response to inflammatory stimuli compared to those from subcutaneous fat and that AKR1B1 may have a predominant role in PGF_2*α*_ synthesis by human preadipocytes [8].

Taking into consideration that excess visceral adipose tissue accumulation is associated with altered metabolic risk independent of total body fat mass [23], depot-specific expression of key enzymes involved in cyclooxygenase-dependent pathways of PG synthesis in adipose tissue may play a pathophysiological role in the development of visceral obesity-associated alterations. PGs seem to alter adipose tissue function by acting as modulators of PPAR*γ* functions [10] and may be responsible for impairing fat storage in abdominal obesity.* In vitro* studies indicated that PGE_2_ and PGF_2*α*_ inhibit the early phase of preadipocyte differentiation by binding to their specific receptors, the EP4 receptor [24] and the FP receptor [25–28], respectively, while others demonstrated that PGD_2_ enhances preadipocyte differentiation [29, 30]. Quinkler et al. previously performed a detailed examination of depot-specific mRNA expression patterns of enzymes involved in PGD_2_ and PGJ_2_ synthesis in human omental and subcutaneous adipose tissues [31] and suggested that these enzymes may have an important role in body fat distribution. However, whether expression of adipose tissue PG-synthesizing enzymes and PG receptors is affected in human obesity and during preadipocyte differentiation has never been clearly established.

Hence, the first aim of this study was to examine depot-specific PGE_2_ and PGF_2*α*_ release and mRNA expression patterns of phospholipase A2 (PLA2G16 and PLA2G4), COX-1, COX-2, PTGDS, PTGIS, PTGFS (AKR1B1), PTGES, PTGES2, PTGES3, prostaglandin FP receptor (PTGFR), and prostaglandin E receptors (PTGER1, PTGER2, PTGER3, and PTGER4) in preadipocytes and whole adipose tissues. The second objective was to identify changes in the expression of these enzymes through preadipocyte differentiation. The third objective was to examine associations between whole adipose tissue mRNA expression of these genes coding for key enzymes or receptors involved in PG synthesis or signaling and measures of adiposity, body fat distribution, or adipocyte size. We tested the hypothesis that cells from the omental fat compartment release more antiadipogenic PGF_2*α*_ and PGE_2_ than those from the subcutaneous depot and that corresponding transcripts show consistent depot differences and modulation through adipogenesis. We also hypothesized that abdominal obesity is related to altered expression of adipose tissue PG-synthesizing enzymes and/or PG receptors.

## 2. Materials and Methods

### 2.1. Participants

The study sample included 46 healthy women (age: 37.6–54.5 years) recruited through the elective surgery schedule of the Gynecology Unit at CHU de Québec Medical Center. Women of the study underwent total (*n* = 45) or subtotal (*n* = 1) abdominal hysterectomies, sometimes accompanied by salpingo-oophorectomy of one (*n* = 6) or two (*n* = 12) ovaries. A few weeks before surgery and on the morning of surgery, detailed information was obtained on medication use and reproductive, menstrual, and medical history for each patient. Women using medication affecting metabolic parameters (beta-blockers, ACE inhibitors, fibric acid derivatives, and statins) were not included in the present study. Women reporting use of nonsteroidal anti-inflammatory medication a few weeks before the surgery were also excluded. To perform differentiation, explants, and primary preadipocyte experiments, a similar subsample of women undergoing gynecological surgery was recruited (age: 37.8–57.5 years, BMI: 19.6–41.1 kg/m^2^). Primary preadipocytes were also obtained commercially (*n* = 1 for SC, from a 43-year-old woman) (Zen Bio, NC, USA). The study was approved by the Research Ethics Committees of CHU de Québec Medical Center (Protocol C09-08-086). We also included cultures from women undergoing bariatric surgery (biliopancreatic diversion or sleeve gastrectomy) for the treatment of severe obesity (*n* = 8, age: 26–54 years, and BMI: 40.0–52.7 kg/m^2^) with approval from the Research Ethics Committees of the Quebec Cardiology and Pulmonology Institute (Protocol CER-IUCPQ 21049). All subjects provided written informed consent before their inclusion in the study.

### 2.2. Body Fatness and Body Fat Distribution Measurements

These measurements were performed on the morning of or a few days before surgery. Body composition was assessed by dual-energy x-ray absorptiometry using a Hologic QDR-4500A densitometer and the whole-body software body fan V8.269:3 (Hologic Inc., Bedford, MA, USA). Measurement of abdominal subcutaneous and visceral adipose tissue cross-sectional areas at the L4-L5 vertebrae was performed by computed tomography as previously described [32, 33].

### 2.3. Adipose Tissue Sampling

Subcutaneous adipose tissue was collected at the site of surgical incision (lower abdomen) and omental adipose tissue was collected at the distal portion of the greater omentum. Adipose tissue samples were immediately carried to the laboratory. A portion of the fresh sample was used to perform adipocyte and preadipocyte isolation and a portion of fresh adipose tissue (30 mg) was cut into 5–10 mg pieces and placed in serum-free Medium 199. Adipose tissue explants were kept in culture at 37°C under 5% CO_2_ atmosphere. The remaining portion of the sample was immediately frozen for RNA isolation and expression measurements.

### 2.4. Adipocyte Isolation and Adipocyte Size Measurement

A portion of each fresh tissue sample was digested with type I collagenase in Krebs-Ringer-Henseleit (KRH) buffer according to a modified version of the Rodbell method [34]. Following digestion, cell suspensions were filtered through nylon mesh and mature adipocytes were separated from the stromal-vascular fraction by floatation. Mature cells were washed 3 times with KRH buffer. For cell size measurements, mature adipocyte suspensions were visualized using a phase contrast microscope attached to a camera and computer interface. Pictures of the suspensions were taken and the Scion Image software (Scion Corporation, Frederick, MA, USA) was used to measure the diameter of 250 adipocytes for each tissue sample. Average adipocyte diameter was used in analyses.

### 2.5. Preadipocyte Isolation and Primary Cultures

Preadipocytes were isolated from the stromal-vascular fraction using a modification of the Van Harmelen method [35]. The residual KRH buffer of adipocyte isolation, which contained the stromal-vascular fraction, was centrifuged and the pellet was washed in DMEM-F12 culture medium supplemented with 10% calf serum, 2.5 *μ*g/mL amphotericin B, and 50 *μ*g/mL gentamicin. Stromal-vascular cells were then filtered through 140 *μ*m nylon mesh to remove endothelial/mesothelial cells, placed in culture plates and cultured at 37°C under a 5% CO_2_ atmosphere. Medium was changed every 2-3 days.

### 2.6. Induction of Adipocyte Differentiation

Preadipocytes were seeded in 12-well plates to obtain full confluence within 48 h. Differentiation of fully confluent preadipocyte cultures was induced using standardized differentiation medium and protocols for 0–8 days (Zen Bio, Durham, NC, USA). Differentiation medium consisted of DMEM-F12 supplemented with a PPAR*γ* agonist, insulin, dexamethasone, and 3-isobutyl-1-methylxanthine. To assess the extent of differentiation, we measured mRNA expression of PPAR*γ*. The accumulation of lipid droplets in differentiated cells (after 8 days) was also assessed by Oil Red O-staining. Cells were washed with PBS and fixed with formalin for 1 h. They were then incubated for 2 h with a 4.9 mM Oil Red O solution and washed three times with ddH_2_O. Pictures of differentiated cells were taken using phase contrast microscopy at 20x magnification.

### 2.7. PGE_2_ and PGF_2*α*_ Measurements

PGE_2_ and PGF_2*α*_ release by subcutaneous and omental primary preadipocytes was measured in cells incubated for 24 hours at 37°C in DMEM-F12 medium. PGE_2_ and PGF_2*α*_ release by subcutaneous and omental primary organ cultures was also measured in serum-free Medium 199 incubated for 24 hours at 37°C with the tissue samples. The incubation time was established according to a time-course experiment. PGE_2_ and PGF_2*α*_ release during various stages of omental or subcutaneous preadipocyte differentiation (0, 6 h, 1 day, and 8 days) was also measured in the differentiation medium. On the basis of these measurements, omental and subcutaneous PGE_2_ and PGF_2*α*_ production rate was calculated. PGE_2_ and PGF_2*α*_ content in the media was measured by enzyme immunoassay and acetylcholinesterase-linked PGE_2_ and PGF_2*α*_ tracer (Cayman Chemicals, Ann Arbor, MI, USA) as previously described [36]. Considering the nature and cultivability of primary preadipocytes and adipose tissue explants, PGE_2_ and PGF_2*α*_ release by cultured primary preadipocytes was expressed as pg/mL∗*μ*g protein∗24 h and PGE_2_ and PGF_2*α*_ release by adipose tissue explants was expressed as pg/mL∗mg tissue∗24 h. Omental and subcutaneous PGE_2_ and PGF_2*α*_ production rate during differentiation was expressed as pg/mL∗*μ*g protein∗hour.

### 2.8. Messenger RNA Expression by Quantitative Real-Time PCR

Total RNA was isolated from whole subcutaneous and omental adipose tissue or from primary differentiated and nondifferentiated cultures using the RNeasy lipid tissue extraction kit and on-column DNase treatment (Qiagen, Hilden, DE) following the manufacturer's recommendations. RNA quality and concentration were assessed using the Agilent Technologies 2100 bioanalyzer (Agilent, Santa Clara, CA, USA). Complementary DNA was generated from total RNA using random hexamers, oligo dT_18_, and Superscript III RNase H-RT (Invitrogen Life Technologies, Burlington, ON, Canada) and purified with QIAquick PCR Purification Kit (Qiagen, Hilden, DE). Real-time cDNA amplification was performed in duplicate using the LightCycler 480 (Roche Diagnostics, Indianapolis, IN, USA) and the SYBRGreen I Master (Roche Diagnostics, Indianapolis, IN, USA). The conditions for PCR reactions were as follows: 45 cycles, denaturation at 95°C for 10 sec, annealing at 60°C for 10 sec, and elongation at 72°C for 14 sec, and then reading at 74°C for 5 sec. A melting curve was performed to assess nonspecific signal. Calculation of copy number for each transcript was performed according to Luu-The et al. [37] using the second derivative method and a standard curve of Cp versus logarithm of the quantity. The standard curve was established using known amounts of purified PCR products and a LightCycler 480 v1.5 program provided by the manufacturer (Roche Diagnostics, Mannheim, DE). PCR amplification efficiency was verified. Target gene amplifications were normalized using housekeeping gene expression levels of ATP synthase O subunit (*ATP5O*) for whole tissue extracts or glucose-6-phosphate dehydrogenase (*G6PD*) for preadipocyte differentiation. Expression levels of* ATP5O* were not different in omental versus subcutaneous adipose tissue in our study sample and were used to normalize whole tissue expression levels.* G6PD* mRNA expression was not significantly modulated during preadipocyte differentiation and was used as control in these experiments. Primer sequences were designed using Gene Tools 2.0 software (Biotools Inc., Edmonton, AB, Canada) and their specificity was verified by blast in the GenBank database. The synthesis was performed by IDT (Integrated DNA Technology, Coralville, IA, USA). The transcripts examined were phospholipase A2 (PLA2G16 and PLA2G4), cyclooxygenases 1 and 2 (PTGS1 and PTGS2), PGF synthase aldo-keto reductase 1B1 (AKR1B1), PGI synthase (PTGIS), prostaglandin D synthase (PTGDS), prostaglandin E synthases (PTGES, PTGES2, and PTGES3), PGE receptors 1, 2, 3, and 4 (PTGER1, PTGER2, PTGER3, and PTGER4), and prostaglandin FP receptor (PTGFR). Primer sequences are listed in Supplemental Table 1, see Supplementary Materials available online at http://dx.doi.org/10.1155/2014/451620. Quantitative real-time PCR measurements were performed by the CHU de Québec Research Center Gene Expression Platform (Québec, QC, Canada).

### 2.9. Statistical Analyses

Student's *t*-tests were performed to examine depot differences in mean basal PGE_2_ and PGF_2*α*_ release by primary preadipocytes or adipose tissue explants, mRNA expression levels of the transcripts examined in cultured preadipocytes, and mean PGE_2_ and PGF_2*α*_ production rate during preadipocyte differentiation. To analyze significant changes among scheduled endpoints (0, 6, 24, and 192 h after induction of differentiation) for two depots, three experimental factors were defined: patient donor (random factor), depot, and measurements at the four time points (fixed factors). The latter was analyzed as a repeated-measure factor with the use of an unstructured covariance matrix. The interaction between depot and the scheduled endpoints was considered. Missing values were not imputed. We used residual maximum likelihood as the method of estimation and the Kenward-Roger method to estimate denominator degrees of freedom for the test of fixed effect. The univariate normality assumption was verified with the Shapiro-Wilk tests on the error distribution from the Cholesky factorization of the statistical model. The Brown and Forsythe variation of Levene's test statistic was used to verify homogeneity of variances. Student's paired *t*-tests were performed to assess depot differences in adipose tissue mRNA expression levels of the transcripts examined in whole adipose tissue. Pearson correlation coefficients were computed to quantify associations between adipose tissue mRNA expression of the transcripts and adiposity measurements. Non-normally distributed variables were log- or boxcox-transformed. All data are presented as mean ± SEM. Statistical analyses were performed with the statistical packages R v3.0.2. (R Foundation for Statistical Computing, Vienna, Austria) and JMP software version 4.0 or SAS v9.4 (SAS Institute Inc, Cary, NC, USA).

## 3. Results 

### 3.1. PGE_2_ and PGF_2*α*_ Release by Adipose Tissue Explants and Primary Preadipocytes from the Subcutaneous and Omental Compartments

**Figure 1:**
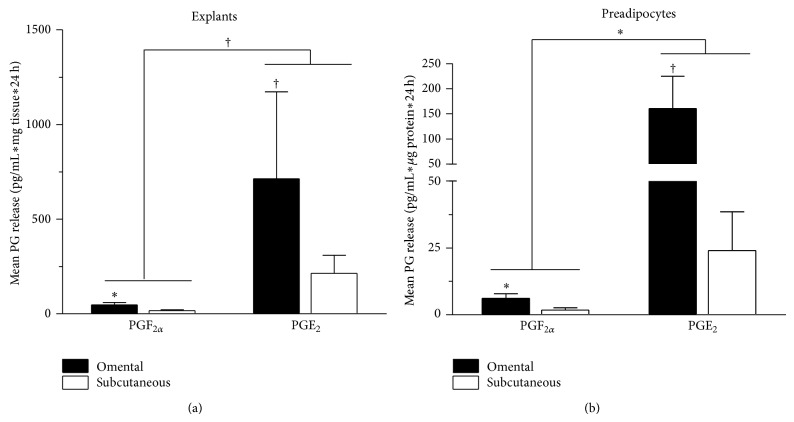
Basal release of PGE_2_ and PGF_2*α*_ by subcutaneous and omental (a) adipose tissue explants (*n* = 8) or (b) primary preadipocyte cultures (OM: *n* = 11 and SC: *n* = 9). PGE_2_ and PGF_2*α*_ release by adipose tissue explants was expressed as pg/mL∗mg tissue∗24 h and PGE_2_ and PGF_2*α*_ release by cultured primary preadipocytes was expressed as pg/mL∗*μ*g protein∗24 h. Results are expressed as mean ± SEM. ^†^ *P* < 0.10; ^*^ *P* ≤ 0.05.

Figure 1 shows PGE_2_ and PGF_2*α*_ release by subcutaneous and omental explants or primary preadipocyte cultures over 24 hours. Adipose tissue explants tended to release more PGE_2_ than PGF_2*α*_ in both adipose tissue compartments (*P* ≤ 0.10) (Figure 1(a)). The release of PGF_2*α*_ by omental explants was significantly higher compared to that of subcutaneous adipose tissue explants (*P* ≤ 0.05) (Figure 1(a)). Omental explants also tended to have a higher PGE_2_ release compared to subcutaneous explants (*P* ≤ 0.10).  Figure 1(b) shows that preadipocyte cultures released more PGE_2_ than PGF_2*α*_ in both adipose tissue compartments (*P* ≤ 0.05). Similar to adipose tissue explants, the release of PGF_2*α*_ by omental preadipocytes was significantly higher compared to that of subcutaneous preadipocytes (*P* ≤ 0.05) (Figure 1(b)). Omental preadipocytes also tended to release more PGE_2_ compared to subcutaneous preadipocytes (*P* ≤ 0.10).

### 3.2. Expression Levels of Genes Related to PG Synthesis or Signaling in Whole Adipose Tissue and Primary Preadipocytes from the Subcutaneous and Omental Compartments

Considering that omental PGE_2_ and PGF_2*α*_ release by primary preadipocytes and adipose tissue explants was higher compared to subcutaneous fat cells, we also examined depot differences in adipose tissue expression of several genes coding for enzymes involved in PG synthesis or PG receptors. Messenger RNA abundance of these genes in whole adipose tissues was assessed in 46 women.  Table 1 shows characteristics of this sample. According to the mean BMI (28.0 kg/m^2^) and body composition measurements, women were overweight and covered a wide range of adiposity values.

**Figure 2:**
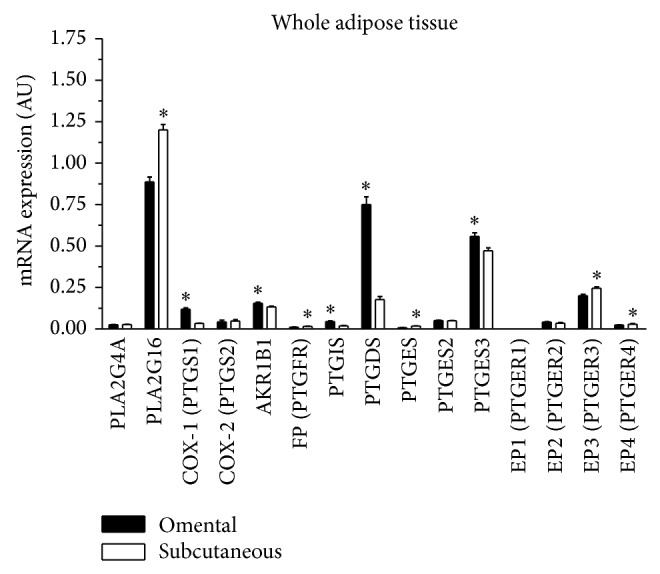
Omental and subcutaneous adipose tissue mRNA expression of several transcripts coding for PG receptors or enzymes involved in PG synthesis in women (*n* = 46). Expression levels relative to ATP5O mRNA abundance are shown. PLA2G4 (phospholipase A2), PLA2G16 (phospholipase A2), PTGS1 (cyclooxygenase 1), PTGS2 (cyclooxygenase 2), AKR1B1 (PGF synthase), PTGFR (prostaglandin FP receptor), PTGIS (PGI synthase), PTGDS (PGD synthase), PTGES, PTGES2, and PTGES3 (PGE synthase), and PTGER1, PTGER2, PTGER3, and PTGER4 (prostaglandin E receptors). Mean ± SEM are shown. AU: arbitrary units ^*^ *P* ≤ 0.05.

The transcripts examined were detectable in whole tissues from both fat compartments and most showed significant depot differences (Figure 2). Specifically, COX-1, AKR1B1, PTGIS, PTGDS, PTGES3, and PTGER1 mRNA expression levels were significantly higher in omental compared to subcutaneous adipose tissue (*P* ≤ 0.05 for all). Expression levels of PLA2G16, PTGFR, PTGES, PTGER3, and PTGER4 were significantly higher in subcutaneous compared to omental adipose tissue (*P* ≤ 0.05 for all). No significant depot difference was observed in PLA2G4, COX-2, PTGES2, and PTGER2 expression levels. PTGER1 mRNA abundance was weak in whole tissues (i.e., below 2000 copies per *μ*g of total RNA).

**Figure 3:**
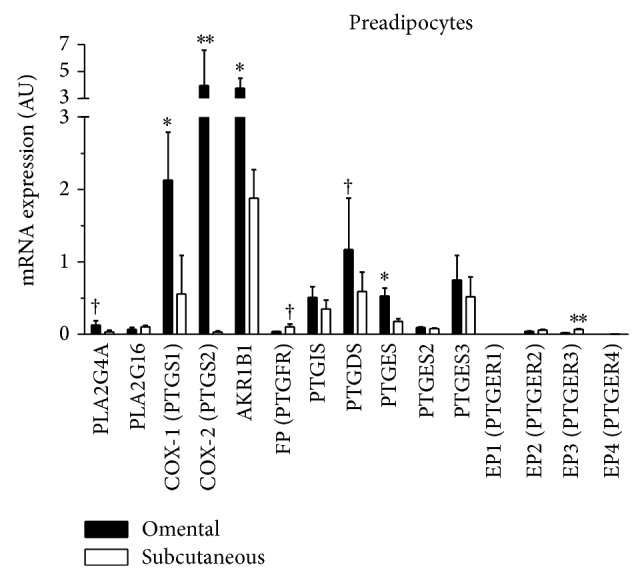
Omental (*n* = 4) and subcutaneous (*n* = 5) preadipocyte mRNA expression of several transcripts coding for PG receptors or enzymes involved in PG synthesis. Expression levels relative to G6PD mRNA abundance are shown. PLA2G4 (phospholipase A2), PLA2G16 (phospholipase A2), PTGS1 (cyclooxygenase 1), PTGS2 (cyclooxygenase 2), AKR1B1 (PGF synthase), PTGFR (prostaglandin FP receptor), PTGIS (PGI synthase), PTGDS (PGD synthase), PTGES, PTGES2, and PTGES3 (PGE synthase), and PTGER1, PTGER2, PTGER3, and PTGER4 (prostaglandin E receptors). Mean ± SEM are shown. AU: arbitrary units ^†^ *P* < 0.10; ^*^ *P* ≤ 0.05; ^**^ *P* ≤ 0.01.

In a subsample of patients, expression of these transcripts was also examined in cultured primary preadipocytes. The transcripts studied were detectable in primary preadipocytes from both fat compartments and most showed significant depot differences (Figure 3). Expression levels of COX-1, COX-2, AKR1B1, and PTGES were significantly higher in omental compared to subcutaneous primary preadipocytes (*P* ≤ 0.05 for all). A trend for higher expression of PLA2G4A and PTGDS in omental preadipocytes was observed (*P* ≤ 0.10 for both). Messenger RNA expression of PTGER3 was significantly higher in subcutaneous compared to omental preadipocytes (*P* ≤ 0.001). A trend for higher expression of PTGFR in subcutaneous preadipocytes was observed (*P* ≤ 0.10). No significant depot difference was observed in PLA2G16, PTGIS, PTGES2, PTGES3, PTGER1, and PTGER2 mRNA expression levels. Messenger RNA expression levels of PTGER1 and PTGER4 were very low in preadipocytes (i.e., below 2500 copies per *μ*g total RNA for PTGER1 and below 300 copies per *μ*g of total RNA for PTGER4).

### 3.3. Expression Levels of PG Enzyme- or Receptor-Coding Genes during Preadipocyte Differentiation

**Figure 4:**
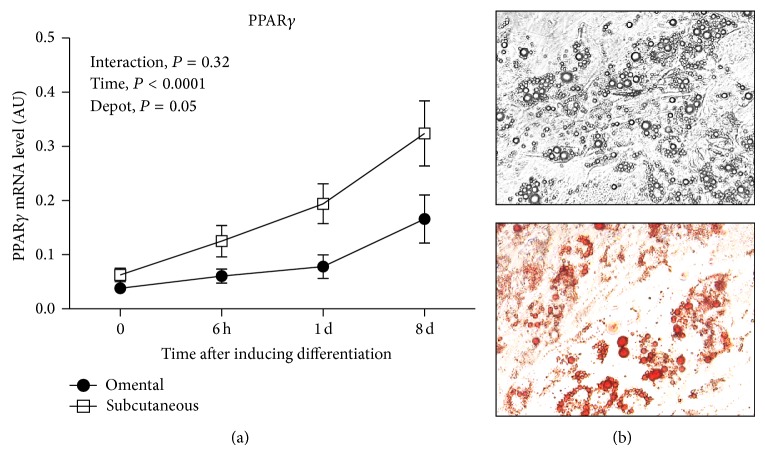
(a) PPAR*γ* mRNA expression during omental (*n* = 4) or subcutaneous (*n* = 5) preadipocyte differentiation (0 h, 6 h, 1 d, and 8 d); (b) eight days after induction of differentiation, lipid storage was assessed using phase contrast microscopy at 20x magnification imaging of differentiated, Oil Red O-stained cells. These pictures were obtained in a representative subcutaneous culture. Expression levels relative to G6PD mRNA abundance. Mean ± SEM are shown. All variables were log-transformed to stabilize variances in statistical analyses. Reported *P* values are based on transformed variables.

To examine whether expression of adipose tissue PG-synthesizing enzymes and PG receptors is modulated during preadipocyte differentiation, we measured these transcripts at 0 h, 6 h, 1 day, and 8 days after induction of differentiation. As expected, Figure 4(a) shows that mRNA expression of PPAR*γ* was significantly induced in differentiated cells from both fat compartments compared to undifferentiated cells, as expected from treatments (*P* < 0.0001). Consistent with these results, the accumulation of lipid droplets in differentiated cells (after 8 days) was also observed by Oil Red O-staining and phase contrast microscopy (Figure 4(b)). Subcutaneous PPAR*γ* mRNA abundance was significantly higher at all time points tested compared to omental PPAR*γ* mRNA abundance, with a significant depot effect (*P* = 0.05) (Figure 4(a)).

**Figure 5:**
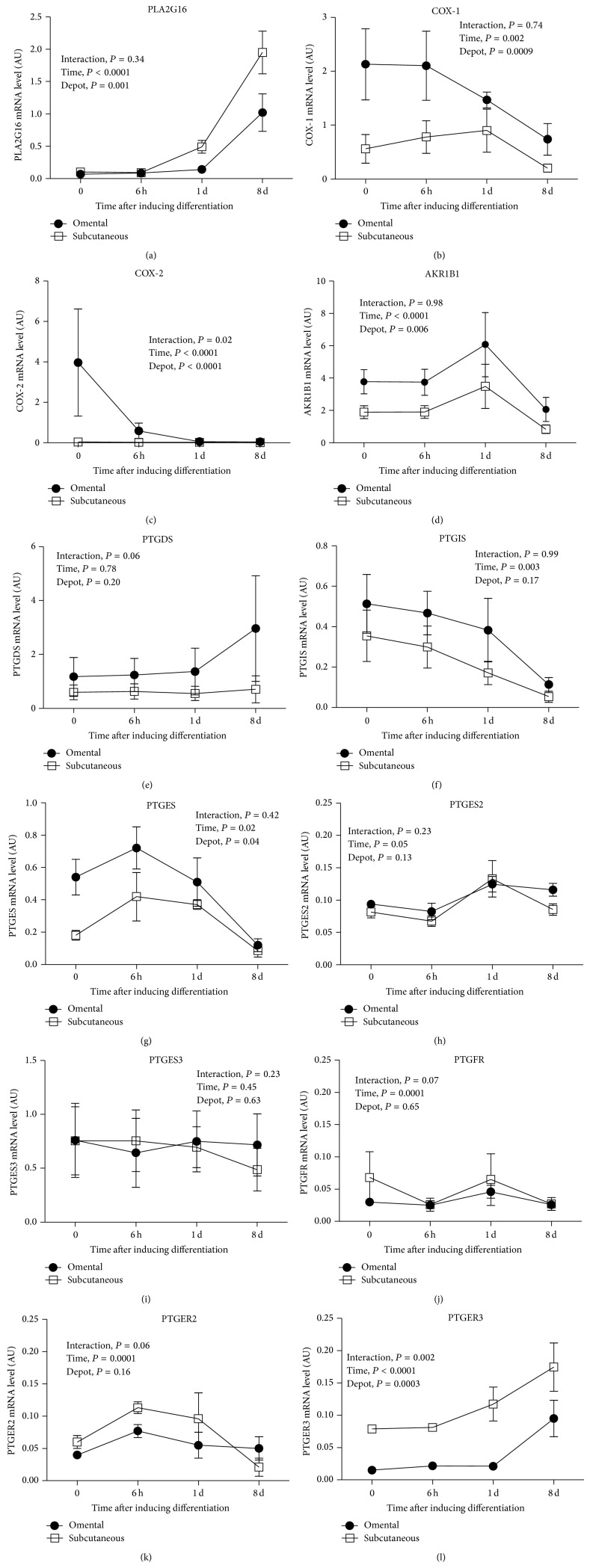
(a) PLA2G16, (b) COX-1 (PTGS1), (c) COX-2 (PTGS2), (d) AKR1B1, (e) PTGDS, (f) PTGIS, (g) PTGES, (h) PTGES2, (i) PTGES3 (j) PTGFR, (k) PTGER2, and (l) PTGER3 mRNA expression during omental (*n* = 4) or subcutaneous (*n* = 5) preadipocyte differentiation (0 h, 6 h, 1 d, and 8 d). Expression levels relative to G6PD mRNA abundance. Mean ± SEM are shown. All variables were log-transformed to stabilize variances. Reported *P* values are based on transformed values. AU: arbitrary units.

Eight days after the induction of preadipocyte differentiation, PLA2G16 and PTGER3 mRNA expression in both fat compartments were significantly increased (Figures 5(a) and 5(l)) whereas COX-1, COX-2, PTGIS, and PTGES mRNA abundance in both fat depots were decreased (*P* ≤ 0.05 for all) (Figures 5(b), 5(c), 5(f), and 5(g)). Interestingly, omental COX-2 mRNA was highly abundant before differentiation and significantly decreased 6 hours after induction, with a significant time-by-depot interaction (*P* = 0.02) (Figure 5(c)). Higher expression of AKR1B1 mRNA in both fat compartments was observed one day after induction of differentiation (*P* < 0.0001) (Figure 5(d)), but this transcript was significantly lower in differentiated cells (after 8 days) compared to undifferentiated cells (*P* = 0.01). A similar pattern of expression was observed for PTGFR. This transcript was slightly decreased after 6 hours, especially in the subcutaneous depot, but significantly increased after one day in both depots (*P* = 0.0001) (Figure 5(j)). PTGDS mRNA expression tended to increase in omental differentiated cells only (time-by-depot interaction, *P* = 0.06) (Figure 5(e)). PTGES2 mRNA expression in both fat compartments was significantly increased after one day compared to undifferentiated cells (*P* = 0.05) (Figure 5(h)). No significant difference was observed in PGTES3 mRNA expression during differentiation (Figure 5(i)). PTGER2 mRNA expression in both fat compartments was significantly increased 6 hours after the induction of differentiation, but this transcript was decreased in differentiated cells, especially in the subcutaneous depot (time-by-depot interaction, *P* = 0.06) (Figure 5(k)). PTGER1 and PTGER4 were only weakly expressed in undifferentiated and differentiated cells (i.e., below 2000 copies per *μ*g of total RNA for PTGER1 and below 1000 copies per *μ*g of total RNA for PTGER4). The level of PTGER1 was gradually decreased after 8 days of differentiation in both fat compartments (*P* = 0.006, data not shown). Significantly lower expression of PTGER4 was observed 6 hours after the induction of differentiation compared to control in both fat compartments (*P* = 0.006). However, this transcript tended to be higher in differentiated cells compared to control (*P* = 0.09, data not shown).

Depot differences in these transcripts were also observed during preadipocyte differentiation. More specifically, subcutaneous PLA2G16 mRNA expression was significantly higher compared to omental PLA2G16 mRNA expression (*P* = 0.001) (Figure 5(a)). Omental COX-1, COX-2, AKR1B1, and PTGES mRNA expression were also significantly higher compared to those of subcutaneous cells (*P* ≤ 0.05 for all) (Figures 5(b), 5(c), 5(d), and 5(g)). Subcutaneous PTGER3 mRNA abundance was significantly higher compared to omental PTGER3 mRNA expression after induction of differentiation (*P* = 0.003) (Figure 5(l)). No significant depot difference was observed for mRNA abundance of PTGDS, PTGIS, PTGES2, PTGES3, PTGFR, and PTGER2 during the induction of preadipocyte differentiation (Figures 5(e), 5(f), 5(h), 5(i), 5(j), and 5(k)).

**Figure 6:**
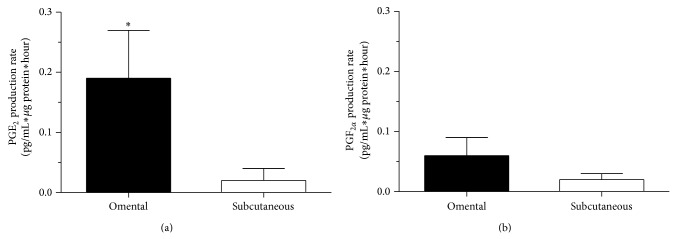
PGE_2_ (a) and PGF_2*α*_ (b) production rate during omental (*n* = 4) or subcutaneous (*n* = 5) preadipocyte differentiation (0 h to 8 days). PGs production rate was expressed as pg/mL∗*μ*g protein∗hour. Mean ± SEM are shown. ^*^ *P* ≤ 0.05.

PGE_2_ and PGF_2*α*_ release by these cells was also measured in the medium at 0, 6 h, 1 day, and 8 days after the induction of differentiation. To examine depot differences, omental and subcutaneous PGE_2_ and PGF_2*α*_ production rate was calculated on the basis of these measurements. Omental PGE_2_ secretion rate was significantly higher compared to subcutaneous PGE_2_ secretion rate (*P* = 0.04, Figure 6(a)). Furthermore, omental PGF_2*α*_ secretion rate tended to be higher compared to subcutaneous PGF_2*α*_ secretion rate (*P* = 0.14, Figure 6(b)).

### 3.4. Whole Adipose Tissue Expression Levels of PG Enzyme- or Receptor-Coding Genes in relation to Body Fatness and Body Fat Distribution

Associations between expression levels of PG enzyme- or receptor-coding genes in whole adipose tissue and body composition or fat distribution measurements was performed to test the hypothesis that abdominal obesity is related to altered expression of adipose tissue PG-synthesizing enzymes and/or PG receptors and that the transcripts that are stimulated through adipogenesis are those that correlate best with the degree of obesity.  Table 2 shows Pearson correlation coefficients between mRNA expression levels of several transcripts examined and body composition or fat distribution measurements. PLA2G16 mRNA expression in omental adipose tissue was positively and significantly correlated with total adipose tissue area and adipocyte diameters (*P* ≤ 0.05 for all). AKR1B1 mRNA expression in both fat compartments was positively and significantly associated with lean body mass (*P* ≤ 0.05). Expression of this transcript in subcutaneous adipose tissue was also positively and significantly correlated with total body fat mass, adipose tissue areas, and adipocyte sizes (*P* ≤ 0.05 for all). PTGDS mRNA expression in omental adipose tissue was positively associated with visceral adipose tissue area (*P* ≤ 0.05). Trends were observed between omental PTGDS mRNA expression and total body fat mass as well as omental adipocyte diameter (*P* ≤ 0.10 for both). Expression of this transcript in subcutaneous adipose tissue was not significantly related to body composition and fat distribution measurements. PTGER3 mRNA expression in omental adipose tissue was positively correlated with total and visceral adipose tissue areas as well as adipocyte sizes (*P* ≤ 0.05 for all). PTGER4 mRNA abundance in both fat depots was associated with body fat distribution indices and omental adipocyte size (*P* ≤ 0.05 for all). PTGER4 mRNA expression in subcutaneous adipose tissue was positively correlated with subcutaneous adipocyte size and body composition (*P* ≤ 0.05 for all). PTGES2 mRNA expression in omental adipose tissue was positively associated only with visceral adipose tissue area (*P* ≤ 0.05, data not shown). No significant associations were found between COX-1, COX-2 PTGIS, PTGES, PGTES3, PTGFR, PTGER1, or PTGER2 mRNA expression in either fat compartment and body composition or fat distribution measurements (data not shown).

## 4. Discussion

To our knowledge, this is the first study to clearly investigate fat depot-specific PGE_2_ and PGF_2*α*_ release combined with expression patterns of several enzymes involved in PG synthesis or signaling in human preadipocytes or whole adipose tissues. In addition, we determined which transcripts were modulated in response to preadipocyte differentiation and we examined the association between body composition or fat distribution measurements and the expression levels of these transcripts in human subcutaneous and omental adipose tissues. We found that cultured preadipocytes and explants from the omental fat compartment release more PGE_2_ and PGF_2*α*_ than those from the subcutaneous depot and the corresponding transcripts show consistent depot differences. During preadipocyte differentiation, PGE_2_ and PGF_2*α*_ secretion rates were higher in omental compared to subcutaneous cells and consistent depot differences in transcripts involved in PGE_2_ and PGF_2*α*_ synthesis were also observed. Transcripts that were stimulated during adipogenesis were those that correlated best with adiposity measurements, suggesting that obesity-related changes in cell number and size affect PG-synthesizing genes and PG receptor expression.

One important finding in this study is that preadipocytes and adipose tissue explants from the omental fat compartment both released more PGE_2_ and PGF_2*α*_ than those from the subcutaneous depot. The findings of higher expression of COX-1, AKR1B1, PTGES3, PTGIS, and PTGDS in omental versus subcutaneous adipose tissue as well as higher expression of COX-1, COX-2, AKR1B1, PTGES, and PTGDS in omental versus subcutaneous cultured preadipocytes are consistent with these results. Several studies previously established that the two COXs are the rate-limiting enzymes in the synthesis of PG [11, 16]. In our study, higher expression of COX isoforms in visceral fat may explain depot differences in PG synthesis. Farb et al. [38] recently demonstrated in severely obese subjects that expression of COXs, PTGES, PTGDS, and PTGIS were upregulated in visceral adipose tissue, which is highly consistent with our results. They also observed that the release of PGE_2_ and 6-keto PGF_1*α*_ was significantly higher in visceral compared to subcutaneous fat after 48 hours of culture [38]. Quinkler et al. also reported that COX-1 and PTGDS mRNA expression levels were higher in omental compared to subcutaneous adipose tissue [31]. Furthermore, we previously found that visceral preadipocytes were more responsive to inflammatory signals in terms of PGF_2*α*_ release [8]. In the present study, our results suggest a predisposition of omental fat cells to release COX-derived prostaglandins, which represents convincing evidence supporting the notion that omental fat is distinct from subcutaneous adipose tissue in terms of endocrine function and that visceral adipose tissue contributes to the inflammatory phenotype [39]. Depot-specific expression patterns of key enzymes involved in PG synthesis or signaling may play a pathophysiological role in visceral obesity. In agreement with this hypothesis, Farb et al. established that PGs of the cyclooxygenase pathway are involved in vascular endothelial dysfunction of visceral adipose tissue [38].

In the present study, we observed that subcutaneous mRNA expression of PPAR*γ* was significantly higher compared to that of the omental fat compartment. In agreement with previous studies regarding depot differences in adipogenesis [40–44], preadipocytes from the subcutaneous fat compartment appeared to have a higher ability to differentiate compared to preadipocytes from visceral fat. Interestingly, during preadipocyte differentiation, we found that the production rate of PGE_2_ and PGF_2*α*_ was lower in the subcutaneous fat compartment and consistent depot differences were also observed in transcripts involved in PGE_2_ and PGF_2*α*_ synthesis (COXs, AKR1B1, and PTGES). These depot differences in PG synthesis during the induction of preadipocyte differentiation are consistent with the notion that PGE_2_ and PGF_2*α*_ may have antiadipogenic functions. Indeed, as mentioned,* in vitro* studies demonstrated that PGE_2_ and PGF_2*α*_ inhibit the early phase of preadipocyte differentiation by acting through their specific receptor [24–28, 45]. Considering the design of our study, we cannot conclude on cause-and-effect relationships. However, our findings do not exclude a potential impact of PGs on adipocyte differentiation. As impaired adipogenesis is a critical factor linking obesity and metabolic complications [40], further studies are necessary to examine how these two PGs might modulate the expandability of adipose tissue in humans.

To our knowledge, this is the first study to clearly report associations between obesity or visceral fat accumulation and the expression of several PG-synthesizing enzymes or PG receptors in human adipose tissues. Many significant correlations were found between expression levels of several transcripts and adiposity measurements. AKR1B1 expression levels in both fat compartments were positively related to measures of total and visceral adiposity. Only omental PTGDS and PLA2G16 expressions were correlated with adiposity indices. Expression levels of the receptors EP3 and EP4 were also positively correlated with body composition and fat distribution indices, especially in the omental compartment for EP3 receptor and in both fat compartments for EP4 receptor. A striking aspect of these results is that all the significant correlations observed were positive. The multiple significant positive correlations with adipocyte size indirectly suggested that expression patterns of PG-synthesizing enzymes or PG receptors in adipose tissue might reflect the proportion of large, mature adipocytes in each adipose tissue sample. In obese individuals, expansion of adipose tissue leads to increased adipocyte size (hypertrophy) and increased adipocyte number (hyperplasia) [4, 5]. We propose that the correlation pattern we describe reflects a modulating effect of obesity on adipose tissue cell composition, which in turn, affects PG-synthesizing enzymes and PG receptors.

Consistent with a modulating effect of obesity on gene expression, transcripts that were significantly correlated with adiposity were also highly expressed in differentiated cells compared to undifferentiated cells. In contrast, most of the enzymes that were not associated to adiposity were weakly expressed in differentiated cells compared to undifferentiated cells. In a similar manner, transcripts that were not modulated during differentiation such as PTGES3 and subcutaneous PTGDS were not significant correlates of body composition or fat distribution. AKR1B1 seems to be an exception to this pattern as its expression was significantly reduced 8 days after differentiation. However, we also found a transient increase in its expression 1 day after differentiation. This could explain the positive associations between AKR1B1 expression in whole tissue and body composition indices in our correlation analysis. In agreement with these results, Fujimori et al. showed that Akr1b3, the murine ortholog of human AKR1B1, was weakly expressed in 3T3-L1 preadipocytes but increased after the initiation of differentiation and then quickly decreased [46]. In the latter study, COX-2 expression was modulated in a similar manner [46]. Our findings are consistent as expression levels of COX-1 and COX-2 decreased during differentiation. Xie et al. also previously observed that both COXs were downregulated after differentiation of 3T3-L1 cells [47]. On the basis of the transient expression of Akr1b3 in differentiating 3T3-L1 cells, Fujimori et al. showed that Akr1b3 is involved in the suppression of the early phase of adipogenesis through PGF_2*α*_ synthesis and signaling through FP receptors [46]. As mentioned, we also reported that AKR1B1 is a functional PG synthase in humans [8, 20, 21]. These findings on AKR1B1 in adipose tissue raise a potential role of this enzyme in the expandability of adipose tissue and the metabolic complications of abdominal obesity.

The expression of PTGES has been previously investigated in adipocytes [45, 48], but the specific isoform involved in the production of PGE_2_ has never been clearly identified in human adipose tissue. Fujimori et al. recently demonstrated that PTGES is responsible for PGE_2_ synthesis in 3T3-L1 cells [16]. Hétu and Riendeau demonstrated that expression of PTGES was greatly decreased in murine obese tissues, whereas PTGES2 and PTGES3 expression showed minor or no changes [48]. Fain et al. also showed that adipocytes isolated from human subcutaneous and visceral adipose tissue of morbidly obese individuals tended to release less PGE_2_ than those from leaner individuals [49]. Our results are not entirely concordant with these previous analyses as we found no correlations between PTGES, PTGES2, or PTGES3 mRNA expression and total adiposity measurements. The fact that we studied women who were in the lean to moderately obese range as opposed to morbidly obese individuals may possibly explain these differences. Xie et al. revealed that PTGES expression is enhanced while level of the cytosolic form (PTGES3) is unchanged during murine 3T3-L1 differentiation [47]. Conversely, Fujimori et al. indicated that the protein and mRNA levels of all three isoforms of PTGES (1, 2, and 3) were constitutively expressed during adipogenesis [16], which is not consistent with our results. Regarding PGE_2_ receptors, we demonstrated that EP1 receptor was weakly expressed in both whole adipose tissue and preadipocytes. We also found that EP4 receptor was rapidly decreased 6 h after the induction of differentiation, which is consistent with a study demonstrating that PGE_2_-EP4 signaling suppresses adipocyte differentiation [16]. Furthermore, we found that the expression of EP3 receptor was significantly higher in differentiated cells, indirectly supporting the notion that EP3 receptor is specific to mature adipocytes [12].

Limitations of the study should be acknowledged. We only have expression data and posttranscriptional or translational modifications may affect protein levels differently. In the context of our study, we examined a rather large sample of patients for which we also had detailed data on obesity levels and adipose tissue distribution as well as visceral/subcutaneous adipose tissue samples. However, we cannot extrapolate our findings to men. Furthermore, we could not determine whether the main PG-secreting cells are in the stromal-vascular fraction of adipose tissue as done by others [50–52]. This is due to the fact that PG release by preadipocytes and adipose tissue explants were not normalized for comparable values. Indeed, PG release by adipose tissue explants was normalized by milligrams of tissue while PG release by adherent preadipocytes in primary cultures was expressed as a function of total protein amount. Additional studies are needed to understand the mechanisms underlying changes in the expression of these enzymes in abdominal obesity and through adipocyte differentiation.

In conclusion, cells from the omental fat compartment release more PGF_2*α*_ and PGE_2_ than those from the subcutaneous depot and the corresponding transcripts show consistent depot differences. Obesity modulates adipose tissue expression of PG-synthesizing enzymes and PG receptors. This likely occurs through adipogenesis-induced changes in expression of these transcripts.

## Supplementary Material

Supplemental Table 1 shows primer sequences of the transcripts examined [phospholipase A2 (PLA2G16 and PLA2G4), cyclooxygenase 1 and 2 (PTGS1 and PTGS2), PGF synthase aldo-keto reductase 1B1 (AKR1B1), PGI synthase (PTGIS), prostaglandin D synthase (PTGDS), prostaglandin E synthases (PTGES, PTGES2 and PTGES3), PGE receptors 1, 2, 3 and 4 (PTGER1, PTGER2, PTGER3 and PTGER4), prostaglandin FP receptor (PTGFR), ATP synthase O subunit (ATP5O) as well as glucose-6-phosphate dehydrogenase (G6PD)].

## Figures and Tables

**Table 1:** Characteristics of the sample (*n* = 46).

Variables	Mean ± SD	Range (min–max)
Age and anthropometrics		
Age (yrs)	46.8 ± 4.0	37.6–54.5
Weight (kg)	73.6 ± 17.0	48.0–133.0
Waist circumference (cm)	93.9 ± 14.7	71.5–147.0
BMI (kg/m²)	28.0 ± 6.4	19.5–50.1
Body composition		
Total body fat mass (kg)^a^	27.4 ± 8.8	10.6–50.4
Lean body mass (kg)^a^	43.6 ± 6.8	29.3–55.3
Adipose tissue areas (cm²)		
Total^c^	407 ± 146	93–773
Subcutaneous^c^	319 ± 120	59–555
Visceral^a^	98 ± 57	35–266
Adipocyte diameter (*μ*m)		
Subcutaneous^b^	101 ± 14	61–131
Omental^b^	87 ± 16	50–121

^a^ *n* = 45, ^b^ *n* = 44, and ^c^ *n* = 43.

**Table 2:** Pearson correlation coefficients between PLA2G16, AKR1B1, PTGDS, PTGER3, or PTGER4 mRNA expression level in subcutaneous (SC) or omental (OM) adipose tissue and body composition or fat distribution (*n* = 46).

Variables	PLA2G16		AKR1B1		PTGDS		PTGER3		PTGER4	
	OM	SC	OM	SC	OM	SC	OM	SC	OM	SC
Body composition										
Total body fat mass^a^	—	—	—	0.39^*^	0.26^†^	—	—	0.26^†^	0.27^†^	0.47^**^
Lean body mass^a^	—	—	0.31^*^	0.38^*^	—	—	—	—	—	0.36^*^
Adipose tissue areas										
Total^c^	0.31^*^	—	—	0.35^*^	—	—	0.35^*^	—	0.41^*^	0.51^**^
Visceral^a^	—	—	0.26^†^	0.41^*^	0.43^*^	—	0.43^*^	—	0.38^*^	0.48^**^
Subcutaneous^c^	0.24^†^	—	—	0.31^*^	—	—	—	—	0.31^*^	0.52^**^
Adipocyte diameter										
Omental^b^	0.31^*^	—	—	0.47^**^	0.25^†^	—	0.51^**^	—	0.33^*^	0.54^**^
Subcutaneous^a^	0.30^*^	—	—	0.37^*^	—	—	0.35^*^	—	—	0.35^*^

PLA2G16: phospholipase A2, AKR1B1: PGF synthase, PTGDS: PGD synthase, PTGER3: PGE receptor, and PTGER4: PGE receptor. Expression levels relative to ATP5O mRNA expression.

^a^ *n* = 45, ^b^ *n* = 44, ^c^ *n* = 43, ^**^ *P* ≤ 0.001, ^*^ *P* ≤ 0.05, ^†^ *P* ≤ 0.10, and (—): no significant association.
