# Supplementary material for: Expression of Genes Related to Prostaglandin Synthesis or Signaling in Human Subcutaneous and Omental Adipose Tissue: Depot Differences and Modulation by Adipogenesis
Source: Mediators Inflamm. 2014 Nov 11;2014:451620. doi: 10.1155/2014/451620 (PMC4244696; doi:10.1155/2014/451620)
Supplement: Supplementary file 1 — Supplemental Table 1 shows primer sequences of the transcripts examined [phospholipase A2 (PLA2G16 and PLA2G4), cyclooxygenase 1 and 2 (PTGS1 and PTGS2), PGF synthase aldo-keto reductase 1B1 (AKR1B1), PGI synthase (PTGIS), prostaglandin D synthase (PTGDS), prostaglandin E synthases (PTGES, PTGES2 and PTGES3), PGE receptors 1, 2, 3 and 4 (PTGER1, PTGER2, PTGER3 and PTGER4), prostaglandin FP receptor (PTGFR), ATP synthase O subunit (ATP5O) as well as glucose-6-phosphate dehydrogenase (G6PD)]. [file 451620.f1.pdf]

SUPPLEMENTAL DATA

Table 1. Oligonucleotides used in realtime RT-PCR quantification

Gene Symbol	Description	GenBank	Oligonucleotide Sequence 5'→ 3' Sense/ Antisense
**PLA2G4**	Phospholipase A2, group IVA	NM_024420	AAGGACGTGCTGGGAAGGTACA/ GGGATACGGCAGGTTAAATGTG
**PLA2G16**	Homo sapiens phospholipase A2, group XVI (PLA2G16)	NM_007069	AGCCTAAGCCTGGAGACCTGAT/ GCCACATCATACAGCAATTCCTTCTT
**PTGS1**	Prostaglandin-endoperoxide synthase 1 (cyclooxygenase 1)	NM_000962	TTGGGGAGAGTATGATAGAGATTG/ CGGAAGGAAACGTAGGGACAG
**PTGS2**	Prostaglandin-endoperoxide synthase 2 (cyclooxygenase 2)	NM_000963	ATGGGTAATGTTATATGTTCTCCTGC/ TGGTGACTGTTTTAATGAGCTCTG
**PTGES**	Prostaglandin E synthase	NM_004878	GGCTATACCTGGGGACTTGATG/ CAGGAATCCAAGGGGCTAAGA
**PTGES2**	Prostaglandin E synthase 2	NM_025072	AGCCTTCCTCGACTTCCATGC/ GGTCTTGAGGGCGCTGATGAT
**PTGES3**	Prostaglandin E synthase 3	NM_006601	CAAGCATAAAAGAACGGACAGATCA/ AATCATCATCTGCTCCATCTACTTC
**PTGIS**	Prostaglandin I2 (prostacyclin) synthase	NM_000961	ATGCCTGCGAGAGACCCTACA/ GCAAGTCACCTCACCTCTCAGTT
**PTGDS**	Prostaglandin D2 synthase	NM_000954	TACAGCTACCGGAGTCCCCAC/ TATCCTCTGTGAAGCCCTGGG
**AKR1B1**	Aldo-keto reductase family 1B1	NM_001628	GATCGCAGCCAAGCACAATAA/ ACAGCTCAACAAGGCACAGAC
**PTGFR**	Prostaglandin F receptor (FP)	NM_000959	CCTTGCCATCGCCATTCTCAT/ CCAGAAAACACCATGCAGATACC
**PTGER1**	Prostaglandin E receptor 1 (EP1)	NM_000955	TCGCTTCGGCCTCCACCTTCT/ CAGCGCCACCAACACCAGCAT
**PTGER2**	Prostaglandin E receptor 2 (EP2)	NM_000956	TGCAACTTCAGTGTCATTCTCAACCT/ GCAGACGGCGAAGGTGATGGT
**PTGER3**	Prostaglandin E receptor 3 (EP3)	NM_198714	CTGGTCTCCGCTCCTGATAAT/ CAGCAGGTAAACCCAAGGATCC
**PTGER4**	Prostaglandin E receptor 4 (EP4)	NM_000958	CGCCGAGATCCAGATGGTCAT/ CGGCAGAAGAGGCATTTGATC
**PPARG**	Peroxisome proliferator-activated receptor gamma	NM_138712	GTCGGATCCACAAAAAAAGTAGAA/ AGCGGGAAGGACTTTATGTATGA
**ATP5O**	ATP synthase, H+ transporting, mitochondrial F1 complex, O subunit	NM_001697	ATTGAAGGTCGCTATGCCACAG/ AACGACTCCTTGGGTATTGCTTAA
**G6PD**	Glucose-6-phosphate dehydrogenase	NM_000402	GATGTCCCCTGTCCCACCAACTCTG/ GCAGGGCATTGAGGTTGGGAG
